# Safety and efficacy of coronary angiography and percutaneous coronary intervention via distal transradial artery access in the anatomical snuffbox: a single-centre prospective cohort study using a propensity score method

**DOI:** 10.1186/s12872-022-02518-8

**Published:** 2022-03-02

**Authors:** Feng Li, Gan-Wei Shi, Xiao-Long Yu, Rui-Xiao Song, Jian-Qiang Xiao, Hao-Min Huang, La-Mei Li, Liu-Yan Zhang, Chun Gong, Gao-Jun Cai

**Affiliations:** 1 Department of Cardiology, Wujin Hospital Affiliated with Jiangsu University, The Wujin Clinical College of Xuzhou Medical University, 2 North Yongning Road, Changzhou, 213017 Jiangsu Province People’s Republic of China; 2 Department of Ultrasonic, Wujin Hospital Affiliated with Jiangsu University, The Wujin Clinical College of Xuzhou Medical University, Changzhou, 213017 Jiangsu Province China

**Keywords:** Coronary atherosclerotic disease, Percutaneous coronary intervention, Distal transradial artery access, Anatomical snuffbox

## Abstract

**Background:**

This study investigated the safety and efficacy of coronary angiography (CAG) and percutaneous coronary intervention (PCI) via distal transradial artery access (d-TRA).

**Methods:**

For this single-centre prospective cohort study, a total of 1066 patients who underwent CAG or PCI procedures from September 2019 to November 2020 were included. Patients were divided into two groups: the d-TRA group (346) and the conventional transradial artery access (c-TRA) group (720) based on access site. A total of 342 pairs of patients were successfully matched using propensity score matching (PSM) for subsequent analysis.

**Results:**

No significant differences in puncture success rate, procedural method, procedural time, sheath size, contrast dosage or fluoroscopy time were noted between the two groups. The puncture time in the d-TRA group was longer than that in the c-TRA group (*P* < 0.01), and the procedure success rate was lower than that in the c-TRA group (90.94% vs. 96.49%, *P* = 0.01). The haemostasis time in the d-TRA group was shorter than that in the c-TRA group (*P* < 0.01), and the visual analogue scale (VAS) was lower than that in the c-TRA group (*P* < 0.01). In addition, the prevalence of bleeding and haematoma in the d-TRA group was lower than that in the c-TRA group (1.75% vs. 7.31%, *P* < 0.01; 0.58% vs. 3.22%, *P* = 0.01, respectively). No significant difference in the incidence of numbness was noted between the two groups. No other complications were found in two groups.

**Conclusion:**

d-TRA is as safe and effective as c-TRA for CAG and PCI. It has the advantages of improved comfort and fewer complications.

*Trail registration* Chinese Clinical Trial Registry, ChiCTR1900026519.

**Supplementary Information:**

The online version contains supplementary material available at 10.1186/s12872-022-02518-8.

## Background

Coronary atherosclerotic disease (CAD) is the most common cause of human death worldwide [1]. Femoral artery access is a classical access route for coronary angiography (CAG) and percutaneous coronary intervention (PCI). However, this rote has some complications, such as bleeding, haematoma and pseudoaneurysm, and the risk of lower limb thrombosis and pulmonary embolism is increasing due to a long bedridden time after the procedure [2]. During the past three decades, conventional transradial artery access (c-TRA) has shown the advantage of few complications and has gradually replaced femoral artery access as the routine for CAG and PCI [3, 4]. However, c-TRA also has some complications, such as radial artery occlusion, vascular injury, spasm, pseudoaneurysm, arteriovenous fistula, access site bleeding and nerve injury [5–8]. In addition, if the puncture of the c-TRA is failure will be crossover to the femoral artery in a non-negligible proportion of cases. However, it is associated with worse prognosis which can be predicted [9, 10]. In 2014, Kaledin et al. [11] described distal transradial artery access (d-TRA) as the default technique for coronary procedures, and Roghani-Dehkordi et al. [12] highlighted the advantages of d-TRA among hand arterial access routes at a Middle Eastern transradial course in 2016. On the basis of those experiences, Kiemeneij [13] promoted left d-TRA in the anatomic snuffbox (AS) for improved procedure ergonomics and patient comfort in right-handed subjects. In recent years, an increasing number of studies have focused on the feasibility and safety of CAG and PCI via d-TRA [14]. However, only a few cohorts or randomized controlled trials have been performed to compare the safety and efficacy of CAG and PCI via d-TRA [15–17].

Here in, a prospective cohort study was performed to investigate the safety and efficacy of CAG and PCI via d-TRA using propensity score matching (PSM) in a Chinese population.

## Methods

### Patient population and study design

A total of 1066 patients who underwent CAG or PCI procedures from September 2019 to November 2020 in Wujin Hospital affiliated with Jiangsu University were included. The exclusion criteria were as follows: (1) patients with acute myocardial infarction undergoing emergency PCI; (2) radial artery pulse that cannot be palpated in the distal or conventional transradial artery; (3) infection in the access site; (4) patients with severe liver/kidney failure or coagulation dysfunction; and (5) patients with symptomatic peripheral vascular disease or Raynaud's syndrome. According to the initial puncture site, the patients were divided into two groups: the d-TRA group (n = 346) and the c-TRA group (n = 720). To eliminate confounding factors, PSM at a 1:1 ratio was used between the two groups. Finally, 342 pairs of patients were successfully matched for subsequent analysis. The study was vetted and approved by the Ethics Committee of Wujin Hospital affiliated with Jiangsu University, and all the patients signed the informed consent form.

### Procedures

**Fig. 1:**
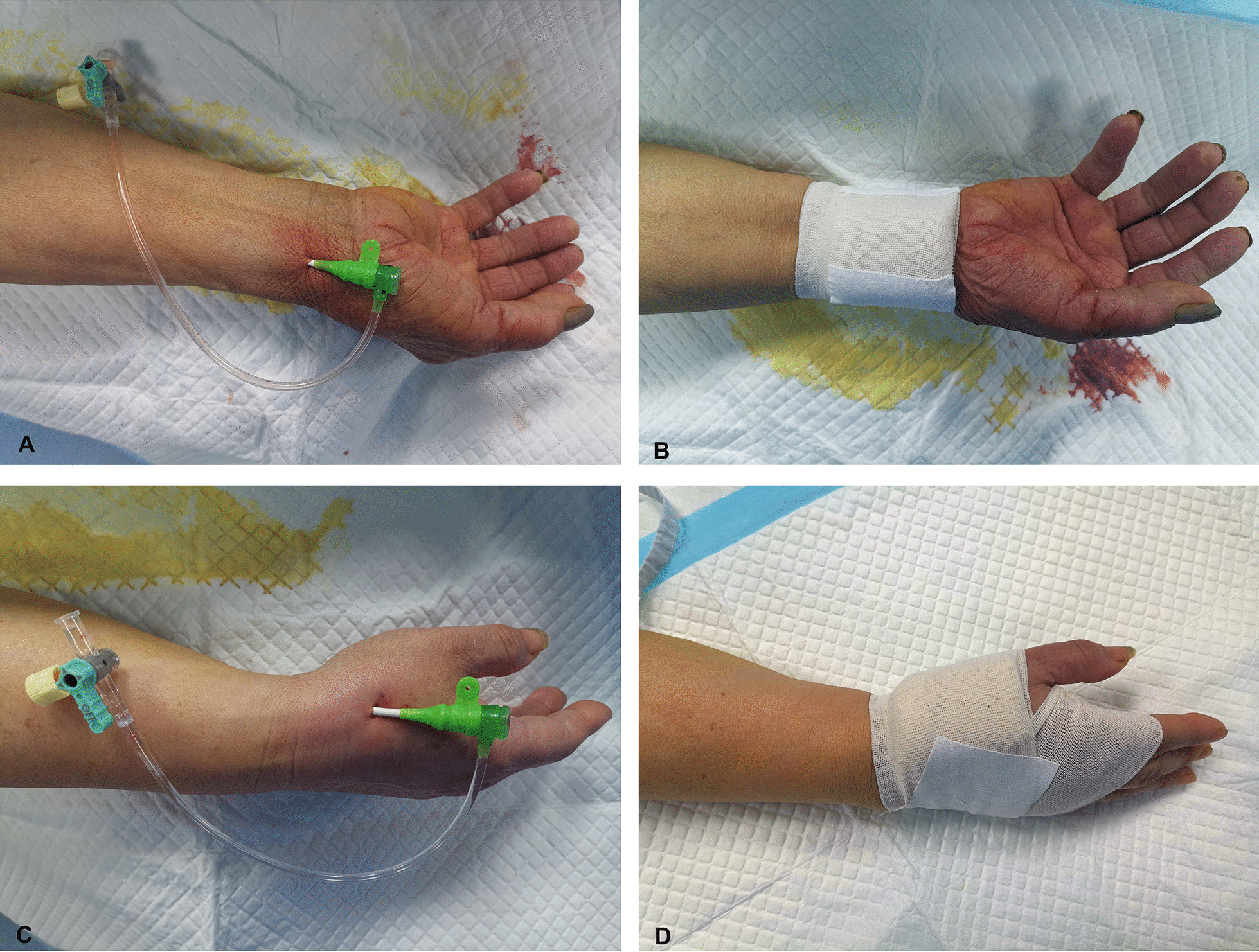
Catheterization by d-TRA and c-TRA. **A** Implantation of the sheath via c-TRA. **B** Haemostasis of the c-TRA. **C** Implantation of the sheath via d-TRA. **D** Haemostasis of the d-TRA

The right hand was the primary access side for the procedures. c-TRA or d-TRA was selected according to operator preference. For the d-TRA group, the access site was in the AS. Accordingly, in the c-TRA group, the access was in the proximal 3 cm of the wrist’s transverse striation. In the d-TRA group, the patient was asked to place the forearm in a natural vertical position and to grasp his thumb under the other four fingers to expose the AS area. For the c-TRA group, the arm abduction was 70°, and the wrist was overextended, which fully exposed the radial artery. Following disinfection, 2% lidocaine was used for local anaesthesia. Then Seldinger’s technique puncture was performed in the AS and the wrist. The puncture was performed with a 20-G puncture needle and a 0.025″ guidewire (Terumo Corporation, Tokyo, Japan, match a 6 French introducer sheath). After a successful puncture, an introducer sheath was placed, and 3000 U heparin and 200 μg nitroglycerine were administered through the side-port of the sheath. Then, a 5 French TIG catheter was used to complete the CAG. In cases of PCI, the procedure was continued after changing the guide catheter. After the procedure, a gauze was used in both the d-TRA and c-TRA groups for haemostasis (Fig. 1). The procedure crossed over to the left d-TRA if the puncture failed in the d-TRA group, and the left c-TRA was used in the c-TRA group.

### Clinical observation index and performance assessment

Patient demographic and clinical characteristics, laboratory examination, and intraprocedural and post procedure data of the two groups were recorded in an electronic database. The data included the following: sex, age, body mass index (BMI), previous medical history, uric acid (UA), creatinine (Cr), glucose (Glu), total cholesterol (TC), triglyceride (TG), low-density lipoprotein cholesterol (LDL-C), high-density lipoprotein cholesterol (HDL-C), glycosylated haemoglobin (HbA1c), puncture time, puncture success rate, procedural time, procedure success rate, contrast dosage, fluoroscopy time, visual analogue scale (VAS), access site with or without haemorrhage and haematoma. If pulsatile swelling or a palpable thrill was noted at the access site after the procedure, an ultrasound examination was used to confirm the diagnosis of arteriovenous fistula or pseudoaneurysm. The outcomes and performance of the procedures were measured as follows: (1) puncture time, time from the beginning of the puncture to the insertion of the artery sheath; (2) puncture success rate determined by arterial blood ejection from the puncture sheath after a successful puncture; (3) procedure success, which is noted when the whole procedure was completed from the same access; (4) haemostasis time, time from the beginning of compression on the access site to the complete decompression; (5) VAS [18] with a 10-cm straight line divided into 10 equal parts to rate pain with 0 indicating no pain and 10 for extreme pain (the patient would note the corresponding position according to the puncture site’s compression and the haemostasis, which determine the patient's pain level); (6) bleeding was classified by the BARC (Bleeding Academic Research Consortium) criteria [19]; and (7) haematoma was assessed according to the EASY (Early Discharge After Transradial Stenting of Coronary Arteries) classification [20].

### Propensity score matching

In this study, to minimize selection bias of the two groups, a 1:1 propensity score matching (PSM) analysis was performed to adjust the baseline difference. Factors, such as age, sex, BMI, previous medical history, UA, CR, Glu, TC, TG, LDL-C, HDL-C, and HbA1c were included in the PSM model using greedy nearest neighbor matching without replacement and with a caliper of 0.01. Standardized differences were evaluated before and after matching to assess the performance of the model. Standardized differences of less than 10.0% indicated a relatively small imbalance.

### Statistical analysis

All the analyses were performed with the statistical software packages R (http://www.R-project.org, The R Foundation) and EmpowerStats (http://www.empowerstats.com, X&Y Solutions, Inc, Boston, MA). Continuous variables with a nonnormal distribution and categorical variables were expressed as medians/quartiles [M/(P25, P75)] and counts or percentages [n (%)], respectively, and the differences were detected with a nonparametric test and chi-square test, respectively. A *P* value < 0.05 was considered statistically significant.

## Results

### Comparison of baseline data before and after matching between the two groups

**Fig. 2:**
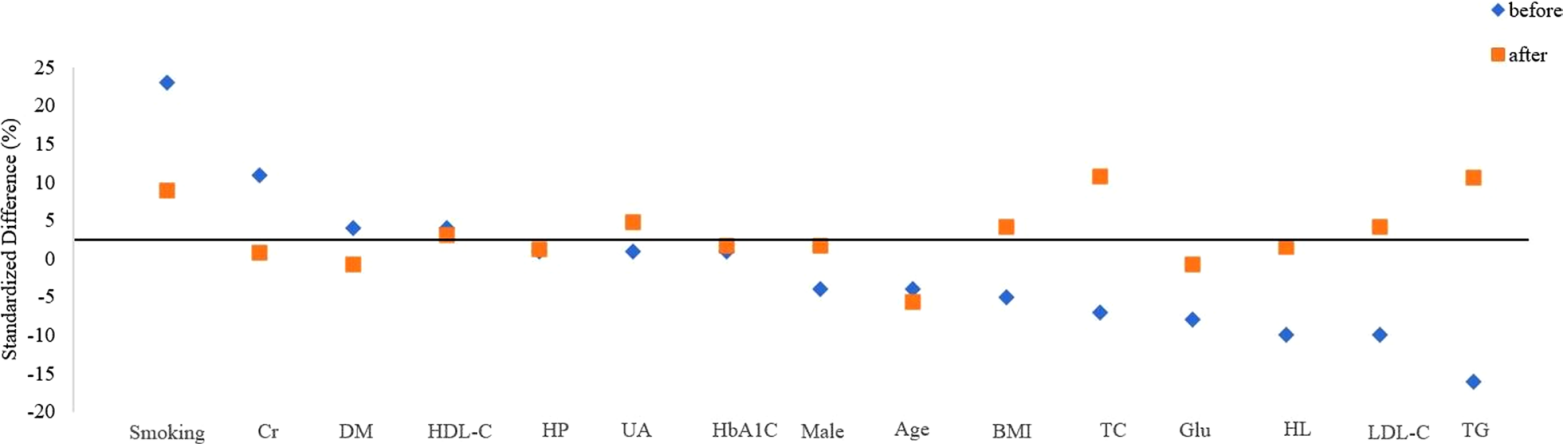
Baseline characteristics before and after propensity score matching

The patient demographic and clinical characteristics are shown in Table 1. Significant differences in the percentage of smoking and TG levels were noted between the two groups before matching. Using PSM, 342 pairs of patients in the d-TRA and TRA groups were successfully matched, and no statistically significant differences in fifteen confounding variables were noted between the two groups (Table 1 and Fig. 2).

**Table 1:** Comparison of baseline data before and after matching between two groups

Characteristic	Before matching		*χ*^2^(*Z*)	*P*	After matching		χ^2^(*Z*)	*P*
	d-TRA (N = 346)	c-TRA (N = 720)			d-TRA (N = 342)	c-TRA (N = 342)		
Male [n (%)]	214 (61.85)	458 (63.61)	0.31	0.58	211 (61.70)	208 (60.80)	0.06	0.81
Age [*M*/(*P*_25_, *P*_75_), yrs]	66.50 (58.00, 72.25)	67.00 (59.00, 73.00)	− 0.51	0.61	66.50 (58.00, 72.00)	67.00 (59.00, 73.00)	− 0.54	0.59
BMI [*M*/(*P*_25_, *P*_75_), kg/m^2^]	24.26 (22.34, 26.69)	24.80 (22.43, 26.87)	− 1.05	0.30	24.26 (22.34, 26.69)	24.45 (22.04, 26.56)	− 0.32	0.75
Smoking [n (%)]	142 (41.04)	216 (30.00)	12.77	< 0.01	139 (40.60)	124 (36.30)	1.39	0.24
EH [n (%)]	248 (71.68)	514 (71.39)	0.01	0.92	245 (71.60)	243 (71.10)	0.03	0.87
DM [n (%)]	94 (27.17)	183 (25.42)	0.37	0.54	91 (26.60)	92 (26.90)	0.01	0.93
HLD [n (%)]	13 (3.76)	42 (5.83)	2.06	0.15	13 (3.80)	12 (3.50)	0.04	0.84
UA [*M*/(*P*_25_, *P*_75_), umol/L]	335.75 (278.63, 403.75)	335.55 (279.30, 404.90)	− 0.13	0.89	335.45 (278.25, 400.55)	332.25 (277.03, 395.05)	− 0.56	0.58
Cr [*M*/(*P*_25_, *P*_75_), µmmol/L]	72.65 (60.00, 83.93)	70.80 (60.20, 81.08)	− 1.28	0.20	72.55 (59.98, 83.83)	71.25 (60.60, 82.70)	− 0.14	0.89
Glu [*M*/(*P*_25_, *P*_75_), mmol/L]	5.18 (4.67, 6.05)	5.22 (4.67, 6.38)	− 1.09	0.28	5.18 (4.67, 6.06)	5.18 (4.64, 6.35)	− 0.45	0.65
TC [*M*/(*P*_25_,*P*_75_), mmol/L]	3.95 (3.20, 4.72)	3.99 (3.24, 4.80)	− 0.64	0.52	3.95 (3.20, 4.72)	3.80 (3.15, 4.66)	− 1.34	0.18
TG [*M*/(*P*_25_,*P*_75_), mmol/L]	1.46 (1.04, 2.03)	1.58 (1.12, 2.32)	− 2.91	< 0.01	1.48 (1.06, 2.03)	1.46 (1.08, 2.07)	− 0.56	0.58
LDL-C [*M*/(*P*_25_,*P*_75_), mmol/L]	2.54 (2.02, 3.18)	2.69 (2.04, 3.33)	− 1.79	0.07	2.56 (2.03, 3.18)	2.60 (1.96, 3.22)	− 0.33	0.74
HDL-C [*M*/(*P*_25_,*P*_75_), mmol/L]	1.09 (0.92, 1.31)	1.06 (0.93, 1.25)	− 0.66	0.51	1.09 (0.92, 1.31)	1.08 (0.93, 1.26)	− 0.35	0.73
HbA1c [*M*/(*P*_25_,*P*_75_),%]	6.10 (5.80, 6.80)	6.00 (5.70, 6.90)	− 1.05	0.30	6.10 (5.80, 6.80)	6.10 (5.70, 7.00)	− 0.40	0.69

*d-TRA* distal transradial artery access, *c-TRA* conventional transradial artery access, *BMI* body mass index, *EH* essential hypertension, *DM* diabetes mellitus, *HLD* hyperlipidemia, *UA* uric acid, *Cr* creatinine, *Glu* glucose, *TC* total cholesterol, *TG* triglyceride, *LDL-C* low-density lipoprotein cholesterol, *HDL-C* high-density lipoprotein cholesterol, *HbA1c* glycated hemoglobin

### Comparison of the effectiveness between the matched groups

No significant differences in sheath size, procedure method, contrast dosage or fluoroscopy time were noted between the two groups (*P* > 0.05). The puncture time in the d-TRA group was longer than that in the c-TRA group [80 (70, 100) s vs. 60 (60, 66) s, *P* < 0.01], and the procedure success rate was lower than that in the c-TRA group (90.94% vs. 96.49%, *P* = 0.01). However, interestingly, the puncture success rate and the procedural time were not significantly different between the two groups (93.57% vs. 96.49%, *P* = 0.15; 35 (20, 60) vs. 30 (20, 60), *P* = 0.37, respectively, Table 2). Among the patients with successful puncture in the d-TRA group, a guide wire could not be inserted into the distal radial artery in seventeen patients. Of these, the sheath was finally inserted with the assistance of the working guide wire (0.014″ Runthrough NS) in eight patients. Patients in the d-TRA group for whom the procedure was not successful all completed the procedure through crossover to the left d-TRA. For the patients in the c-TRA group for whom the procedure was not successful, all procedures were ultimately completed upon crossover to the left c-TRA. No patients required switching to the femoral artery access.

**Table 2:** Comparison of the effectiveness between the matched groups

Characteristic	d-TRA (N = 342)	c-TRA(N = 342)	*P*
Puncture time [*M*/(P_25_, P_75_), s]	80 (70, 100)	60 (60, 66)	< 0.01
Puncture success rate [n (%)]	320 (93.57)	330 (96.49)	0.15
Sheath [n (%)]			0.32
6F	341 (99.71)	339 (99.12)	
7F	1 (0.29)	3 (0.88)	
Procedural method [n (%)]			0.18
CAG	219 (64.04)	202 (59.06)	
PCI	123 (35.96)	140 (40.94)	
CAD [n (%)]	250 (73.10)	275 (80.41)	0.02
Number of diseased vessels [n (%)]			0.23
Single	72 (28.80)	97 (35.27)	
Double	90 (36.00)	84 (30.55)	
Multiple	88 (35.20)	94 (34.18)	
Procedural time [*M*/(P_25_, P_75_), min]	35 (20, 60)	30 (20, 60)	0.37
Procedure success rate [n (%)]	311 (90.94)	330 (96.49)	0.01
Contrast dosage [*M*/(P_25_, P_75_), ml]	80 (50, 150)	70 (50, 150)	0.59
Fluoroscopy time [*M*/(P_25_, P_75_), min]	7.54 (3.17, 15.11)	6.21 (2.47, 14.14)	0.07

*CAG* coronary angiography, *PCI* percutaneous coronary intervention, *CAD* coronary atherosclerotic disease

Subgroup analysis of the d-TRA group stratified by sex, age, BMI, essential hypertension (EH), diabetes mellitus (DM) and smoking showed that female patients, nonsmoking patients, patients with EH and without DM had a higher puncture success rate, which increased gradually with age and BMI. However, no statistically significant differences were noted among the different stratification factors (Additional file 1: Fig. S1).

### Comparison of the safety between the matched groups

The haemostasis time in the d-TRA group was shorter than that in the c-TRA group [4 (3, 6) h vs. 6 (6, 8) h, *P* < 0.01], and the VAS was lower than that in the c-TRA group [3 (2, 3) vs. 4 (3, 5), *P* < 0.01]. All patients with bleeding in both groups were BARC type II, and haematoma was classified as EASY type I. The incidence of bleeding and haematoma in the d-TRA group was lower than that in the c-TRA group (1.75% vs. 7.31%, *P* < 0.01; 0.58% vs. 3.22%, *P* = 0.01, respectively). Further analysis found that bleeding and haematoma were significantly different in CAG patients (bleeding: 0.91% vs. 5.94%, *P* < 0.01; haematoma: 0.00% vs. 2.48%, *P* = 0.02) but not in PCI patients (bleeding: 3.25% vs. 9.29%, *P* = 0.05; haematoma: 1.63% vs. 4.29%, *P* = 0.21). No significant difference in the incidence of numbness was noted between the two groups, and no other complications, such as pseudoaneurysm, arteriovenous fistula, and access site infection, were found (Table 3**)**.

**Table 3:** Comparison of the safety between the matched groups

Characteristic	d-TRA (N = 342)	c-TRA (N = 342)	*P*
Haemostasis time [*M*/(P_25_, P_75_), h]	4 (3, 6)	6 (6, 8)	< 0.01
VAS [*M*/(P_25_, P_75_)]	3 (2, 3)	4 (3, 5)	< 0.01
Bleeding (BARC II) [n (%)]	6 (1.75)	25 (7.31)	< 0.01
CAG	2 (0.91)	12 (5.94)	< 0.01
PCI	4 (3.25)	13 (9.29)	0.05
Haematoma (EASY I) [n (%)]	2 (0.58)	11 (3.22)	0.01
CAG	0 (0.00)	5 (2.48)	0.02
PCI	2 (1.63)	6 (4.29)	0.21
Numbness [n (%)]	3 (0.88)	4 (1.17)	0.70
Pseudoaneurysm [n (%)]	0 (0%)	0 (0%)	1.00
Arteriovenous fistula [n (%)]	0 (0%)	0 (0%)	1.00
Infection [n (%)]	0 (0%)	0 (0%)	1.00

*VAS* visual analogue scale, *BARC* Bleeding Academic Research Consortium, *EASY* Early Discharge After Transradial Stenting of Coronary Arteries

## Discussion

The AS, a triangular-shaped space located on the lateral side of the wrist’s dorsum, is clearly exposed when the thumb is stretched out. The AS is bound by the tendons of the extensor pollicis brevis and the abductor pollicis longus on the lateral side and the tendon of the extensor pollicis longus on the medial side. The base of the triangle is formed by the styloid process of the radius. The trapezium and scaphoid bones composed the bottom of the AS. In the AS region, the radial artery is easily palpated and becomes the best site for puncture given its superficial position and bony basement.

The distal radial artery was first used by anaesthesiologists for intraoperative blood pressure monitoring [21]. Since Kiemenij [13] promoted the left d-TRA in 2017, an increasing number of cardiologic interventionalists have begun to pay attention to this access site [14, 22]. Currently, the puncture and procedure success rates of d-TRA range from 70 to 100%, and most of them are lower than those of c-TRA [14]. The preliminary experience of 34 patients in our centre showed that the puncture and procedure success rates were 91.18% and 85.29%, respectively. Kiemeneij [13] reported that the procedure success rate was 88.57%. Early clinical experience by Valsecchi et al. [23] showed that the overall feasibility was 90.39%. A prospective observational study conducted in a Korean population showed that the puncture success rate was 95.5% [24]. Koutouzis et al. [17] used a small sample randomized controlled study, and found that the puncture time of the d-TRA was longer than that of the c-TRA, but no significant difference in the total procedural time was noted between the two groups. The puncture time in the d-TRA group in this study was longer than that in the c-TRA group [80 (70, 100) s vs. 60 (60, 66) s, *P* < 0.01]. The procedure success rate in the d-TRA group was significantly lower than that in the c-TRA group (90.94% vs. 96.49%, *P* = 0.01). No significant differences in the puncture success rate, procedural method, procedural time, contrast dosage, or fluoroscopy time were noted between the two groups.

The reasons why the procedure success rate in the d-TRA was lower than that in the c-TRA may be explained the following aspects: (1) the diameter of the radial artery in the AS is smaller than that in the wrist [25], which makes it difficult to puncture or to insert the sheath after successful puncture; (2) tortuosity of the distal radial artery commonly exists, which easily leads to the failure of inserting the guidewire and sheath into the radial artery; and (3) for CAG and PCI, d-TRA is a new access that has recently emerged. Most operators lack puncture experience and need to overcome the learning curve.

Previous studies have found that the haemostasis time in d-TRA patients was significantly shorter than that in c-TRA patients [13, 16, 17]. Moreover, because compression in the distal radial artery does not block venous reflux of the hand, swelling, pain, and numbness of the hand do not easily occur. Thus, the patients are comfortable and the procedure is well tolerated [26]. Amin et al. [27] used the VAS to evaluate the degree of pain during haemostasis and found that the pain scores in the d-TRA group were significantly lower than those in the c-TRA group. AI-Azizi et al. [28] found that d-TRA not only improved patient satisfaction but also improved the satisfaction of operators and nurses. In this study, the time of haemostasis in the d-TRA group was significantly shorter than that in the c-TRA group [4 (3, 6) h vs. 6 (6, 8) h, *P* < 0.01], and the VAS was lower than that in the c-TRA group [3 (2, 3) vs. 4 (3, 5), *P* < 0.01]. These features, greatly reduce the nursing workload postprocedure and improve patient comfort and satisfaction.

d-TRA has very few complications, such as haematoma, haemorrhage, pseudoaneurysm and arteriovenous fistula [24, 28], which may be related to the AS with a bony basement surrounded by tendons. In this study, the rates of bleeding and haematoma in the d-TRA group were significantly lower than those in the c-TRA group. Further analysis found a significant difference in bleeding and haematoma in CAG patients, but not in PCI patients. This finding was due to the longer compression time in PCI patients compared with CAG patients in both the d-TRA group and the c-TRA group.

PSM can make the nonrandom data of two groups more similar and achieve covariate balance between the two groups, so the relationship between the research variables and the results can be better obtained [29]. In the present study, PSM was used to eliminate confounding bias and improve statistical effectiveness, which is one of the highlights of this study.

### Study limitations

The study also has some limitations: (1) This study was a single-center prospective cohort study with a small sample size. Although PSM was adopted to eliminate confounding bias to the maximum extent, it could also make both groups so homogenous that differences may be underappreciated in the statistical analysis. These findings still needs to be verified by multicentre, large-sample, randomized controlled clinical trials. (2) Vascular ultrasound was not used to evaluate distal radial artery diameter, radial artery occlusion, pseudoaneurysm, or arteriovenous fistula in all patients. (3) Long long-term follow-up after the procedure is needed.

## Conclusions

d-TRA is as safe and effective as c-TRA for CAG and PCI. It has the advantages of improved comfort and fewer complications.

## Supplementary Information

**Additional file 1.** Subgroup analysis of the puncture success rate in the d-TRA group stratified by sex, age, BMI, EH, DM, and smoking.

## Data Availability

The datasets used and/or analysed during the current study are available from the corresponding author on reasonable request.

## Abbreviations

CAG: Coronary angiography
PCI: Percutaneous coronary intervention
d-TRA: Distal transradial artery access
c-TRA: Conventional transradial artery access
PSM: Propensity score matching
VAS: Visual analogue scale
CAD: Coronary atherosclerotic disease
AS: Anatomic snuffbox
