# Supplementary material for: Safety and efficacy of coronary angiography and percutaneous coronary intervention via distal transradial artery access in the anatomical snuffbox: a single-centre prospective cohort study using a propensity score method
Source: BMC Cardiovasc Disord. 2022 Mar 2;22:74. doi: 10.1186/s12872-022-02518-8 (PMC8892764; doi:10.1186/s12872-022-02518-8)
Supplement: Supplementary file 1 — Additional file 1. Subgroup analysis of the puncture success rate in the d-TRA group stratified by sex, age, BMI, EH, DM, and smoking. [file 12872_2022_2518_MOESM1_ESM.docx]

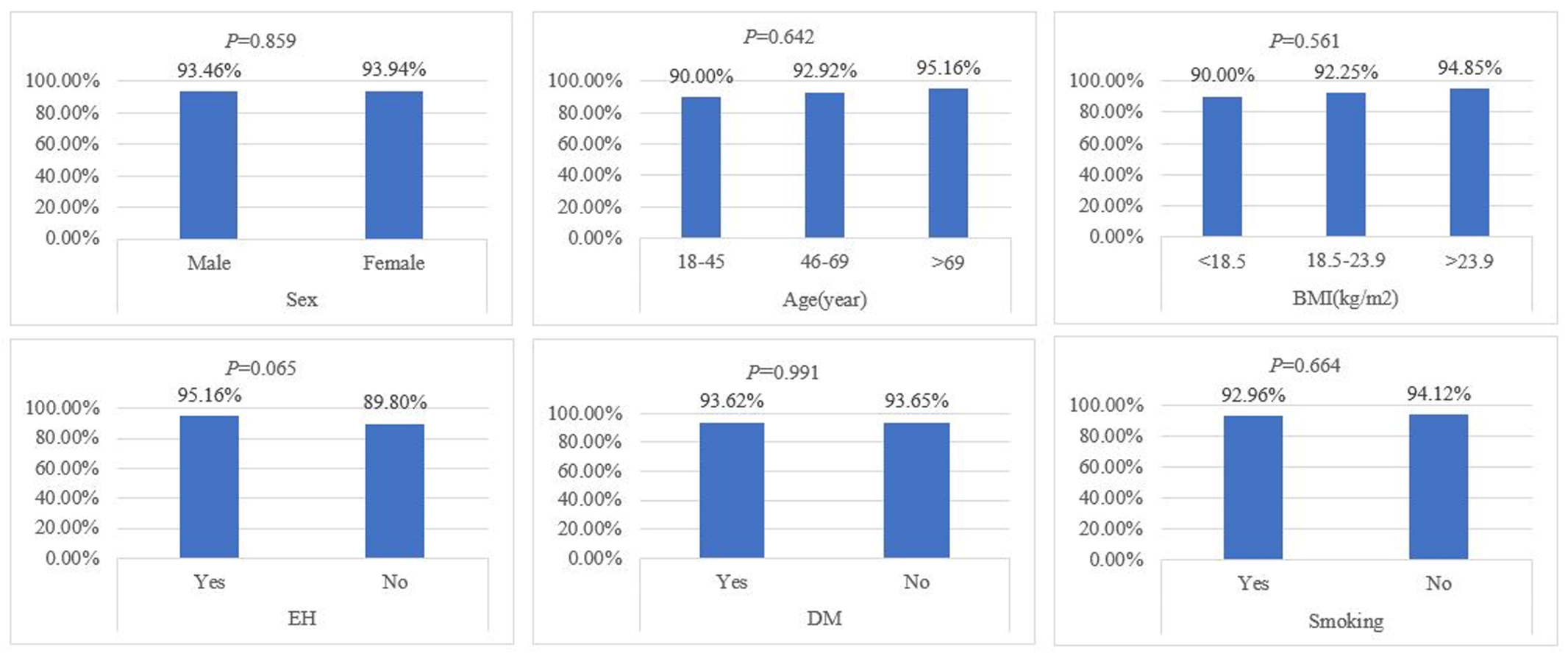


**Supplementary Figure.** Subgroup analysis of the puncture success rate in the d-TRA group stratified by sex, age, BMI, EH, DM and smoking. Age: 18-45 = youth, 46-69 = middle age, > 69 = old age; BMI = body mass index: < 18.5 = underweight, 18.5-23.9 = normal, >23.9 = overweight; EH = essential hypertension; DM = diabetes mellitus.
